# Water sorption, solubility, and relative deformation of thermoplastic materials used for clear aligners: An in vitro study

**DOI:** 10.4317/jced.64490

**Published:** 2026-09-15

**Authors:** Pablo Lopez-Beraun, Carlos Liñan-Duran, Kelly Achachao-Almerco

**Affiliations:** 1 School of Stomatology. Universidad Peruana Cayetano Heredia, Lima, Perú

**Keywords:** Orthodontic appliances removable, absorption, solubility, in vitro techniques (MeSH).

## Abstract

**Background:**

Thermoplastic materials used in the fabrication of clear aligners are continuously exposed to the oral environment; therefore, properties such as water sorption, solubility, and dimensional stability may influence their clinical performance. The objective is to compare, through an in vitro study, the physical properties of thermoplastic materials used for the fabrication of orthodontic aligners, under non-thermoformed and thermoformed conditions, after immersion in distilled water.

**Materials and Methods:**

A total of 104 specimens obtained from four commercial thermoplastic materials: Claris, Pro Aligner X, Taglus Premium, and Essix ACE® were evaluated under non-thermoformed and thermoformed conditions (n=13 per group). Water sorption and solubility were determined according to ISO 20795-2 after immersion in distilled water at 37°C for seven days. Relative deformation was calculated from changes in thickness before and after immersion. Data were analyzed using one-way ANOVA or the Kruskal-Wallis test, according to data distribution (α=0.05).

**Results:**

Significant differences in water sorption were observed among commercial brands in both non-thermoformed (p=0.008) and thermoformed samples (p<0.001). Thermoformed Claris showed the highest water sorption value (22.48 ± 3.20 μg/mm^3^). Solubility showed no significant differences among materials under any experimental condition (p>0.05). Relative deformation differed significantly among brands in both non-thermoformed and thermoformed samples (p<0.001), with the highest value recorded for thermoformed Claris (1.70 ± 0.79%).

**Conclusions:**

The evaluated thermoplastic materials showed different behaviors in water sorption and relative deformation after immersion in distilled water. Thermoformed Claris showed the highest water sorption and relative deformation values, suggesting greater dimensional susceptibility under the evaluated experimental conditions.

## Introduction

Over the last decades, orthodontics has incorporated esthetic alternatives to conventional appliances, including clear aligners, driven by esthetic demand and technological advancement [1-4]. Clear aligners constitute a therapeutic modality based on a sequence of removable thermoplastic trays designed to induce progressive tooth movements through periodic replacement during treatment [4]. Several studies have reported advantages associated with this modality, including favorable perceptions in terms of esthetics, comfort, and oral hygiene [4-7]. However, the biomechanical performance of aligners depends not only on treatment planning and patient cooperation, but also on the characteristics of the thermoplastic material used for their fabrication [4,7].

Currently, clear aligners can be manufactured by thermoforming thermoplastic sheets over physical models obtained from digital workflows, which continues to be the most widely used procedure, as well as by direct three-dimensional printing, which is considered an emerging alternative [4]. Various polymers are used for their fabrication, including polyethylene terephthalate (PET), glycol-modified polyethylene terephthalate (PET-G), thermoplastic polyurethanes (TPU), polycarbonate (PC), polypropylene (PP), ethylene-vinyl acetate (PEVA), and different polymer blends [4,8]. These materials can be classified as amorphous or semicrystalline according to their molecular structure, a condition that directly influences their properties [8].

Exposure of thermoplastic sheets to aqueous media promotes water sorption from the first hours of immersion, with a progressive tendency toward saturation [8]. The magnitude of this phenomenon may vary among materials and depend on intrinsic characteristics of the polymer, such as chemical composition, density, crystallinity, and free volume between chains [9-11]. The penetration of water molecules into the polymer matrix may induce plasticization, stress relaxation, alterations in intra- and interchain bonds, modification of free volume, and dimensional changes associated with hygroscopic expansion of the material [8,10]. Additionally, interaction with aqueous media may produce variations in solubility and microstructural alterations with variable behavior among different thermoplastic polymers [9]. Likewise, thermoforming has been reported to increase water sorption and solubility in materials used for clear aligners [10,11].

In this context, the characterization of properties such as water sorption, solubility, and relative deformation is relevant to understanding the physical behavior of thermoplastic materials clinically used in orthodontics. Therefore, the objective of this study was to compare these properties *in vitro* in different thermoplastic materials, under non-thermoformed and thermoformed conditions.

## Materials and Methods

This was an experimental *in vitro* study.

*Sample*

The sample consisted of thermoplastic sheets used for the fabrication of orthodontic aligners, corresponding to four commercial brands: Claris 0.76 mm (PET-G, Morelli, São Paulo, Brazil), Pro Aligner X 0.76 mm (PET-G, Orthometric, São Paulo, Brazil), Taglus Premium 0.76 mm (PET-G, Taglus, Mumbai, India), and Essix ACE® 0.88 mm (copolyester, Dentsply Sirona Deutschland, Bensheim, Germany), all belonging to the same manufacturing batch.

Sheets were evaluated under two independent experimental conditions: Non-thermoformed and thermoformed. The sheets intended for thermoforming were processed using a thermoforming machine (Ministar S, Scheu, Iserlohn, Germany). For the 0.76-mm-thick sheets (Claris, Pro Aligner X, and Taglus Premium), program 123 of the Ministar S unit was used, whereas program 133 was used for the 0.88 mm Essix ACE® sheets. The thermoforming model consisted of a 3D-printed disk measuring 35 mm in radius and 9 mm in height, based on the study by Elkholy *et al*. [12].

Specimens with standardized dimensions of 10 mm × 10 mm were prepared from each sheet using a manual cutting guillotine. The dimensions of the specimens were verified using a digital caliper (Truper, Mexico; resolution 0.01 mm), and each specimen was assigned a numerical code for identification.

The sample size was determined through an a priori statistical power analysis using G*Power software (version 3.1.9.7; Heinrich Heine Universität Düsseldorf, Germany). A large effect size (Cohen’s *f*=0.40), defined based on the results of the pilot study, a significance level of *α*=0.05, and a statistical power of 0.95 were considered. This analysis determined a total sample size of 104 specimens, distributed as 13 specimens per brand in each experimental condition, achieving a statistical power of 0.96.

*Procedures*

Water sorption and solubility (ISO 20795-2) [13].

Specimen conditioning (m1): The specimens were placed in a glass desiccator with silica gel and stored in an incubator at 37 ± 1°C for 23 ± 1 hours. They were then kept at 23 ± 1°C for 60 ± 10 minutes before weighing. Mass measurements were performed using an analytical balance (Faithful, model FA2204N, China; resolution 0.0001 g). The procedure was repeated until a constant mass was obtained, defined as a variation not greater than 0.2 mg between successive weighings (m1).

Immersion in distilled water (m2): The specimens were completely immersed in containers holding 25 mL of distilled water, ensuring full coverage. They were then stored in an incubator at 37 ± 1°C for 7 days ± 2 hours. At the end of this period, the specimens were removed, dried with absorbent paper until free of surface moisture, and weighed 60 ± 10 seconds after removal from the aqueous medium, recording the saturation mass (m2).

Specimen reconditioning (m3): The specimens were again placed in a desiccator with silica gel at 37 ± 1°C, repeating the procedure described for initial conditioning until a constant mass was obtained.

Calculation of water sorption (Wsp): Water sorption was defined as the amount of water absorbed by the thermoplastic material, determined by the increase in mass after immersion in distilled water at 37°C and expressed in relation to the specimen volume (mg/mm³). Water sorption was calculated using the following formula: Wsp = (m2-m3)/V. Where: 

Wsp: Water sorption (mg/mm³).

m2: Water-saturation mass.

m3: Reconditioned mass.

V: Specimen volume.

Calculation of water solubility (Wsl): Water solubility was defined as the amount of material dissolved after immersion in water, determined by the difference between the initial conditioned mass and the reconditioned mass, normalized according to the specimen volume (mg/mm³). Water solubility was calculated using the following formula: Wsl = (m1-m3)/V. 

Where: 

Wsl: Water solubility (mg/mm³).

m1: Initial conditioned mass.

m3: Reconditioned mass.

V: Specimen volume.

Calculation of relative deformation (Def): Relative deformation was defined as the percentage change in the thickness of the thermoplastic material in relation to its initial thickness.

Specimen thickness was measured using a digital micrometer (ACCUD, China; range 0-25 mm, accuracy 0.001 mm), with measurements taken in the central area of each specimen at each recording point. Measurements were performed before the experimental procedure and at the end of the corresponding immersion period.

Relative deformation was calculated using the following formula: Def = [(e0-e1)/e0] *100. 

Where: 

Def: Relative deformation (%).

e0: Initial thickness.

e1: Thickness after the experimental process.

*Statistical analysis*

Jamovi statistical software, version 2.6.44 (The Jamovi Project, Sydney, Australia), was used. A descriptive analysis was performed for the variable physical properties and its dimensions: Water sorption, solubility, and relative deformation, according to commercial brand and experimental condition, non-thermoformed and thermoformed.

Data distribution was assessed using the Shapiro-Wilk normality test. For variables with a normal distribution, one-way analysis of variance (ANOVA) was used, followed by Tukey’s post hoc test. For variables that did not meet the assumptions of normality, the nonparametric Kruskal-Wallis test was used, with pairwise multiple comparisons performed using the Dwass-Steel-Critchlow-Fligner procedure. A statistical significance level of *α*=0.05 was considered.

*Ethical considerations*

This project was approved by the University Directorate for Regulatory Affairs in Research (DUARI) of Universidad Peruana Cayetano Heredia, under document code CAR-DUARI-O-406-25.

## Results

Statistically significant differences in water sorption values were observed among commercial brands in both non-thermoformed samples (*p*=0.008) and thermoformed samples (*p*<0.001). Under the non-thermoformed condition, Taglus Premium and Pro Aligner X showed the highest mean values, whereas under the thermoformed condition, Claris showed significantly higher values than the other brands (Table 1).

No statistically significant differences in solubility values were identified among commercial brands, either in non-thermoformed samples (*p*=0.369) or in thermoformed samples (*p*=0.085) (Table 2).

Regarding relative deformation, statistically significant differences were found among commercial brands in both non-thermoformed samples (*p*<0.001) and thermoformed samples (*p*<0.001). Pro Aligner X showed the highest median under the non-thermoformed condition, whereas Claris showed the highest mean value under the thermoformed condition (Table 3).

## Discussion

This study aimed to compare the physical properties of different thermoplastic materials used in the fabrication of clear aligners, both under non-thermoformed conditions and after thermoforming, following immersion in distilled water for seven days.

Regarding water sorption, differences were observed among commercial brands, with higher values found for Taglus Premium and Pro Aligner X under the non-thermoformed condition, whereas Essix ACE® showed the lowest water uptake. After thermoforming, Claris showed a significant increase in sorption, reaching values higher than those of the other materials. These results suggest that the hygroscopic behavior of aligners depends not only on their polymer classification, but also on the structural characteristics specific to each material. The literature indicates that factors such as chemical composition, molecular weight, density, crystallinity, and free volume influence the diffusion and retention capacity of water within the polymer matrix [8,14]. In this context, it has been shown that variations in the proportion of units derived from ethylene glycol terephthalate and 1,4-cyclohexanedimethanol terephthalate may modify molecular regularity, crystallization capacity, and the structural organization of PET-G polymers [15]. Therefore, materials marketed under the same polymer classification may exhibit different physical properties, even when they share a similar chemical base. Although the specific composition of the evaluated materials was not determined in the present study, the behavior observed in Claris could be related to these differences within the polymer matrix; however, this interpretation should be considered only as a hypothesis, since the chemical composition of the materials was not directly evaluated. 

The most relevant finding for this property was the marked increase in water sorption observed in Claris after thermoforming. This behavior is consistent with studies that have documented increases in water sorption after thermoforming, attributing them to structural modifications capable of facilitating water diffusion into the polymer [11,14,16]. It should be noted that the water sorption recorded in thermoformed Claris (22.48 μg/mm³) was higher than that reported by Albilali *et al*. [14] for other thermoformed thermoplastic materials used in orthodontics, whose values reached up to 19.93 μg/mm³, and also exceeded the maximum values described by Porojan *et al*. [9] for different PET-G materials, which reached up to 14.10 μg/mm³. Proposed mechanisms include changes in molecular organization, the proportion of amorphous and crystalline regions, and the free volume available for water diffusion [8,10,16]. The relevance of this finding lies in the fact that water sorption may induce plasticization, increase polymer chain mobility, and promote hygroscopic expansion phenomena capable of altering the physical stability of the materials [7,8,17-19]. This was particularly consistent with the thermoformed sheets of the Claris brand, which showed the highest relative deformation values, suggesting a possible association between the hygroscopic behavior of the material and its dimensional stability after thermoforming.

In contrast to water sorption, solubility did not show statistically significant differences among the evaluated brands, either before or after thermoforming, suggesting a limited and comparable release of soluble components under the experimental conditions studied. This behavior is consistent with the available evidence, which describes low or even undetectable solubility values in thermoplastic materials used for aligners, suggesting adequate chemical stability of their polymer matrices [8,14]. A relevant finding was the presence of negative solubility values in some non-thermoformed samples, a phenomenon previously described in the literature and attributed to residual water retention within the polymer matrix after reconditioning [11,14]. Similarly, Albilali *et al*. [14] reported solubility values between -3.01 and -0.06 μg/mm³ in non-thermoformed materials evaluated under comparable experimental conditions, while Daniele *et al*. [20] described values close to zero and even negative after immersion in water, attributed to the same phenomenon of residual water retention. These values do not represent a real gain in material, but rather indicate that the amount of retained water was greater than the mass of components released into the medium [14]. This observation helps explain why the differences recorded in water sorption were not necessarily reflected in equivalent differences in solubility, since both phenomena respond to different mechanisms: While water sorption involves water uptake, solubility reflects the release of components from the polymer matrix.

Although some authors have reported increases in solubility after thermoforming due to structural modifications that favor the release of residual components [11], the present study only observed a trend toward higher values in some thermoformed materials, without reaching statistically significant differences among brands. This suggests that the effect of thermoforming on chemical stability was limited compared with its influence on water sorption. From a biological perspective, these findings are important because solubility is an indirect indicator of the potential release of substances from aligners into the oral environment [4,21]. The observed values suggest limited loss of soluble components during the evaluated period; however, this method does not allow identification of the chemical nature of the released substances or determination of the possible presence of microplastics, nanoplastics, or other degradation products. Therefore, complementary studies aimed at the chemical characterization of released compounds, as well as cytotoxicity and cellular biocompatibility assays, are needed.

Regarding relative deformation, significant differences were observed among the evaluated materials both before and after thermoforming. Under the non-thermoformed condition, Pro Aligner X showed the highest deformation values, whereas Claris showed higher values after thermoforming.

The concurrence of greater water sorption and greater relative deformation observed in thermoformed Claris sheets suggests a possible interaction between both phenomena. This interpretation is consistent with the findings of Ryokawa *et al*. [10], who observed an increase in material thickness after water sorption, and with studies attributing hygroscopic expansion, plasticization, and physical degradation of the polymer matrix to water uptake [7,18-20]. Likewise, Tamburrino *et al*. [7] stated that fluid absorption could alter aligner fit as a consequence of dimensional changes in the material. Although the present study did not directly evaluate clinical fit or the expression of orthodontic forces, this approach is relevant because the clinical efficacy of aligners depends not only on the mechanical properties of the material, but also on its geometric configuration and its adaptation to dental surfaces. In this context, the shape-molding effect describes how the intimate adaptation of the aligner to dental anatomy constitutes a determining factor for the generation and transmission of orthodontic forces [22]. Therefore, dimensional changes associated with greater water sorption and relative deformation could compromise aligner adaptation during clinical use. Nevertheless, this interpretation should be considered a hypothesis, since the present study did not evaluate the three-dimensional fit of the aligners or the magnitude of the orthodontic forces generated.

A strength of this study was the comparative evaluation of four commercially available thermoplastic materials for clear aligners, including PET-G materials and one copolyester, both before and after thermoforming. In addition, water sorption, solubility, and relative deformation were analyzed together using standardized methodologies, providing an integrated assessment of properties closely related to the physical stability of these materials. However, the results should be interpreted considering some limitations inherent to the experimental design. The study was conducted *in vitro* using distilled water and flat 10 × 10 mm specimens, conditions that do not fully reproduce the complexity of the oral environment or the actual geometry of a clinical aligner. Likewise, the chemical composition of the materials was assumed according to the information provided by the manufacturers and was not verified using analytical techniques; therefore, explanations related to polymer structure should be interpreted as hypotheses. Similarly, the relative deformation evaluated in this study does not directly represent the clinical fit of the aligners. Consequently, future research should incorporate artificial saliva, thermocycling, mechanical loading, longer aging periods, and physicochemical, mechanical, and biological analyses to allow a more precise understanding of the behavior of these materials and their clinical impact.

## Conclusions

The evaluated thermoplastic materials showed different behaviors in water sorption and relative deformation after immersion in distilled water. Thermoformed Claris showed the highest water sorption and relative deformation values, suggesting greater dimensional susceptibility under the evaluated experimental conditions. In contrast, solubility remained low and showed no significant differences among materials. These findings should be interpreted within the limitations of an *in vitro* model and require complementation with physicochemical, mechanical, and clinical analyses. 

## Figures and Tables

**Table 1:** Table 1: Water sorption values, expressed in μg/mm^3^, of thermoplastic sheets according to commercial brand in non-thermoformed and thermoformed samples.

**Brand**	**Experimental condition**	
	**Non-thermoformed**	**Thermoformed**
	Mean (SD)	Mean (SD)
Taglus Premium	13.27 (4.03)^a^	11.37 (2.78)^a^
Pro Aligner X	13.55 (3.87)^a^	10.64 (2.68)^a^
Claris	9.95 (3.91)^ab^	22.48 (3.20)^b^
Essix ACE	9.15 (3.55)^b^	8.88 (2.82)^a^

Different letters within each experimental condition indicate significant differences among commercial brands according to Tukey’s post hoc test after one-way ANOVA (p<0.05).

**Table 2:** Table 2: Solubility values, expressed in μg/mm^3^, of thermoplastic sheets according to commercial brand in non-thermoformed and thermoformed samples.

**Brand**	**Experimental condition**	
	**Non-thermoformed**	**Thermoformed**
	Mean (SD)	Mean (SD)
Taglus Premium	-0.982 (1.74)	2.97 (2.05)
Pro Aligner X	0.509 (2.59)	2.57 (2.31)
Claris	-0.590 (2.79)	4.23 (3.77)
Essix ACE	-0.166 (1.48)	1.30 (2.94)

No significant differences were observed among commercial brands within each experimental condition according to one-way ANOVA (p>0.05).

**Table 3:** Table 3: Relative deformation percentage (%) of thermoplastic sheets according to commercial brand in non-thermoformed and thermoformed samples after immersion.

**Brand**	**Experimental condition**	
	**Non-thermoformed**	**Thermoformed**
	Median (IQR)	Mean (SD)
Taglus Premium	0.127 (0.128)^a^	0.313 (0.205)^a^
Pro Aligner X	0.399 (0.004)^b^	0.257 (0.212)^a^
Claris	0.382 (0.129)^c^	1.7 (0.794)^b^
Essix ACE	0.11 (0.011)^a^	0.158 (0.095)^a^

Different letters within each column indicate significant differences among commercial brands (p<0.05). Non-thermoformed: Median (IQR); Kruskal-Wallis test with Dwass-Steel-Critchlow-Fligner comparisons. Thermoformed: Mean (SD); one-way ANOVA with Tukey’s test.

## Data Availability

Declared none.
